# ECCO_2_R in 12 COVID-19 ARDS Patients With Extremely Low Compliance and Refractory Hypercapnia

**DOI:** 10.3389/fmed.2021.654658

**Published:** 2021-07-08

**Authors:** Xin Ding, Huan Chen, Hua Zhao, Hongmin Zhang, Huaiwu He, Wei Cheng, Chunyao Wang, Wei Jiang, Jie Ma, Yan Qin, Zhengyin Liu, Jinglan Wang, Xiaowei Yan, Taisheng Li, Xiang Zhou, Yun Long, Shuyang Zhang

**Affiliations:** ^1^Department of Critical Care Medicine, State Key Laboratory of Complex Severe and Rare Diseases, Peking Union Medical College Hospital, Chinese Academy of Medical Science and Peking Union Medical College, Beijing, China; ^2^Department of Medical Intensive Care Unit, State Key Laboratory of Complex Severe and Rare Diseases, Peking Union Medical College Hospital, Chinese Academy of Medical Science and Peking Union Medical College, Beijing, China; ^3^Department of Nephrology, State Key Laboratory of Complex Severe and Rare Diseases, Peking Union Medical College Hospital, Chinese Academy of Medical Science and Peking Union Medical College, Beijing, China; ^4^Department of Infectious Diseases, State Key Laboratory of Complex Severe and Rare Diseases, Peking Union Medical College Hospital, Chinese Academy of Medical Science and Peking Union Medical College, Beijing, China; ^5^Department of Pulmonary and Critical Care Medicine, State Key Laboratory of Complex Severe and Rare Diseases, Peking Union Medical College Hospital, Chinese Academy of Medical Science and Peking Union Medical College, Beijing, China; ^6^Department of Cardiology, State Key Laboratory of Complex Severe and Rare Diseases, Peking Union Medical College Hospital, Chinese Academy of Medical Science and Peking Union Medical College, Beijing, China

**Keywords:** COVID-19, hypercapnia, acute respiratory distress syndrome, extracorporeal carbon dioxide removal, driving pressure

## Abstract

**Purpose:** A phenotype of COVID-19 ARDS patients with extremely low compliance and refractory hypercapnia was found in our ICU. In the context of limited number of ECMO machines, feasibility of a low-flow extracorporeal carbon dioxide removal (ECCO_2_R) based on the renal replacement therapy (RRT) platform in these patients was assessed.

**Methods:** Single-center, prospective study. Refractory hypercapnia patients with COVID-19-associated ARDS were included and divided into the adjusted group and unadjusted group according to the level of PaCO_2_ after the application of the ECCO_2_R system. Ventilation parameters [tidal volume (VT), respiratory rate, and PEEP], platform pressure (Pplat) and driving pressure (DP), respiratory system compliance, arterial blood gases, and ECCO_2_R system characteristics were collected.

**Results:** Twelve patients with refractory hypercapnia were enrolled, and the PaCO_2_ was 64.5 [56-88.75] mmHg. In the adjusted group, VT was significantly reduced from 5.90 ± 0.16 to 5.08 ± 0.43 ml/kg PBW; DP and Pplat were also significantly reduced from 23.5 ± 2.72 mmHg and 29.88 ± 3.04 mmHg to 18.5 ± 2.62 mmHg and 24.75 ± 3.41 mmHg, respectively. In the unadjusted group, PaCO_2_ decreased from 94 [86.25, 100.3] mmHg to 80 [67.50, 85.25] mmHg but with no significant difference, and the DP and Pplat were not decreased after weighing the pros and cons.

**Conclusions:** A low-flow ECCO_2_R system based on the RRT platform enabled CO_2_ removal and could also decrease the DP and Pplat significantly, which provided a new way to treat these COVID-19 ARDS patients with refractory hypercapnia and extremely low compliance.

**Clinical Trial Registration:** https://www.clinicaltrials.gov/, identifier NCT04340414.

## Introduction

Recently, COVID-19 disease caused by the novel coronavirus (SARS-CoV-2) has been a worldwide severe epidemic problem and resulted in thousands of deaths (1). Respiratory manifestation was one of the main clinical characteristics of this disease; about 15-20% of suspected and confirmed patients developed severe hypoxemia and required mechanical ventilation (2). Gattinoni et al. divided the COVID-19 pneumonia into two phenotypes: Type L and Type H (3), but in our clinical practice in Wuhan, we encountered a group of ARDS patients who presented a different phenotype from the two mentioned above, with refractory carbon dioxide (CO_2_) retention, extremely low lung compliance, and low lung recruitability, which was also found in other centers (4, 5). Hypercapnia not only impairs innate immunity *via* evolutionarily conserved mechanisms (6), which reduce the ability to fight infection, but also has hemodynamic consequence, increasing pulmonary hypertension and worsening right ventricular function (7). A recent study showed that severe hypercapnia (PaCO_2_ ≥ 50 mmHg) appeared to be independently associated with higher ICU mortality in patients with ARDS (8). In order to correct the severe hypercapnia, minute ventilation and drive pressure were often forced to increase to far beyond the level of lung protective ventilation. This means higher mechanical energy and a higher risk of ventilator-related lung injury (9, 10). Therefore, extracorporeal carbon-dioxide removal (ECCO_2_R) device came into our consideration. In this sudden outbreak of COVID-19, like all countries in the world (11), the number of ECMO machines has been in a state of serious shortage for quite a long time, and also the specific ECCO_2_R system. However, the RRT device is more feasible, and recent studies had improved that, ECCO_2_R based on a RRT platform enabled very low tidal volume ventilation with moderate increase in PaCO_2_ in patients with ARDS patients (12, 13). Therefore, this prospective study was designed to assess whether the application of the ECCO_2_R system on RRT platform could decrease the DP and Pplat, thereby facilitated the protective ventilation in these patients.

## Methods

### Patients

This single-center, prospective study was conducted during March 7 to April 15 in a newly constructed 32-bed ICU in Wuhan. All the medical staff were from Peking Union Medical College Hospital (PUMCH); 70% of them had experiences in the ICU ward. All the patients admitted were transferred from other hospitals and were all identified with COVID-19. This study was approved by the ethics review board of PUMCH (ZS-2332), and informed consent was obtained from legally authorized surrogates. The clinical trial protocol was registered with www.clinicaltrials.gov/ (Clinicaltrials.gov identifier: NCT04340414).

Refractory hypercapnia patients with COVID-19-associated ARDS were included, if the following inclusion criteria were met: (1) diagnosed with ARDS according to the Berlin definition and lung protective strategy was implemented after admission, which included low tidal volume (VT) ventilation (Vt 6 ml/kg of predicted body weight), low plateau pressure (Pplat <30 cmH_2_O), higher PEEP strategy, and prone positioning 16–20 h per day; (2) evolved into refractory hypercapnia (PaCO_2_ > 50 mmHg), despite efforts of correcting CO_2_ retention by increasing the respiratory rate and driving pressure. The exclusion criteria were patients with ICU stay <24 h, decompensated heart failure, pregnancy, age <18 years, acute brain injury, contradictions of systemic anticoagulation, catheter access to femoral vein or jugular vein impossible, and decision to limit therapeutic interventions.

### ECCO_2_R System

**Figure 1:**
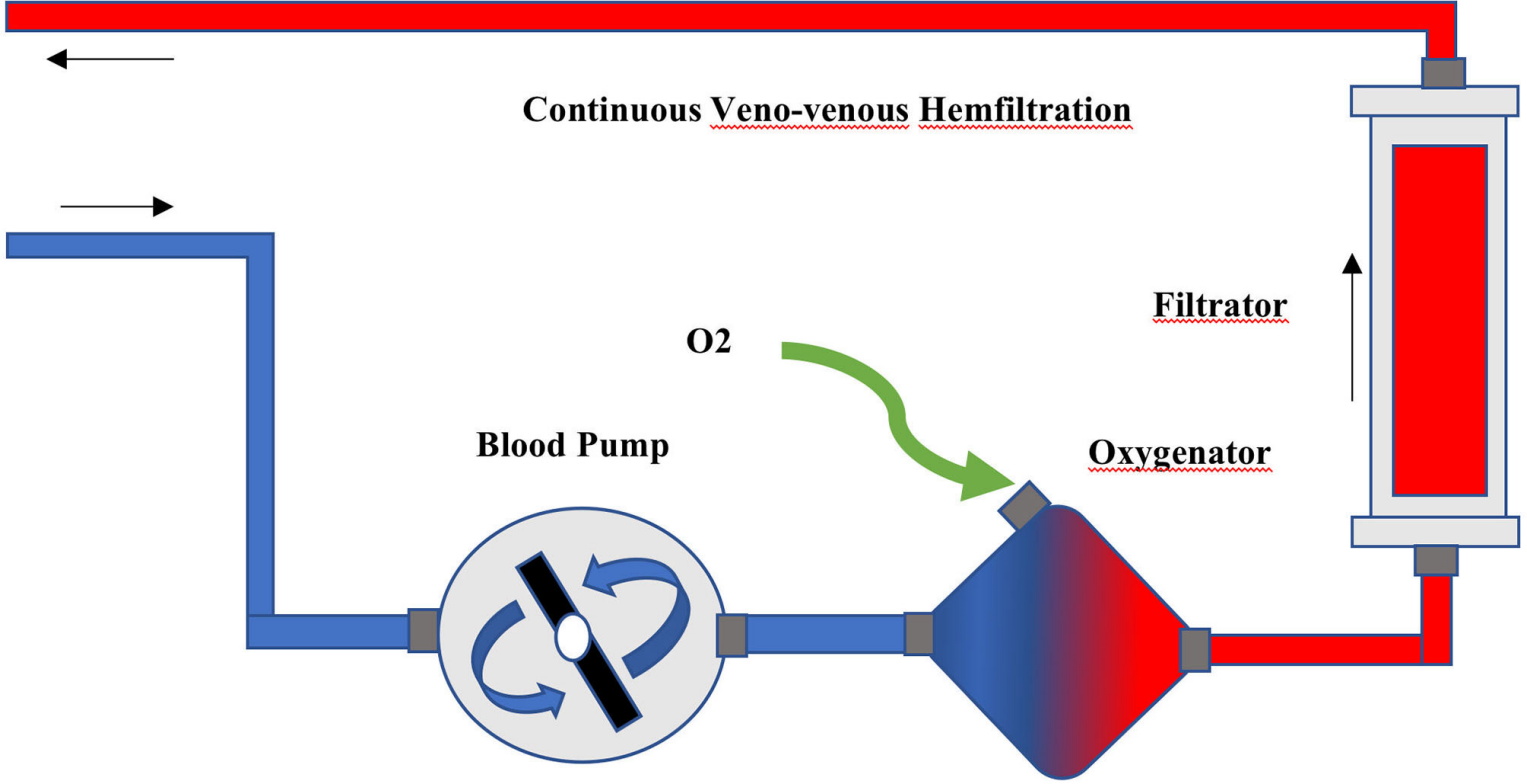
The connection of hemofiltration and extracorporeal oxygenator.

The ECCO_2_R was provided by a low-flow gas-exchanger oxygenator (QUADROX-I pediatric HMO30000, MAQUET) integrated into the Primsaflex platform (Gambro-Baxter) with the slow continuous ultrafiltration (SCUF) mode, and the ultrafiltration was set at 0. The polymethyl pentene, hollow fiber, gas-exchanger membrane was connected to the extracorporeal circuit before the RRT filter (Figure 1). Two 12-Fr two-lumen hemodialysis catheters (arrow) were aseptically and percutaneously inserted under ultrasonography guidance into the right jugular vein and one of the femoral veins with a femoral-jugular pattern to prevent self-recirculation and improve the clearance efficiency. Systemic heparinization was used to maintain the activated partial thromboplastin time ratio (aPTTr) 1.5–2.0 × that of the control. The continuous venous, arterial line and filter pressures were monitored in the Prismaflex device.

### Protocol

After priming, the Prismaflex device was connected to the patient and the extracorporeal blood flow was progressively increased to 300-400 ml/min. In the beginning, a flow test was done to assess the efficiency of CO_2_ clearance of the membranes. Pre- and post-oxygenator blood PCO_2_ were compared when the sweep flow was adjusted to 0, 5, 10, and 15 L/min, and back to 0 L/min. Then, the sweep-gas flow through the ECCO_2_R was switched on the level with the best clearance efficiency. The changes of CO_2_ clearance with time were also collected.

**Figure 2:**
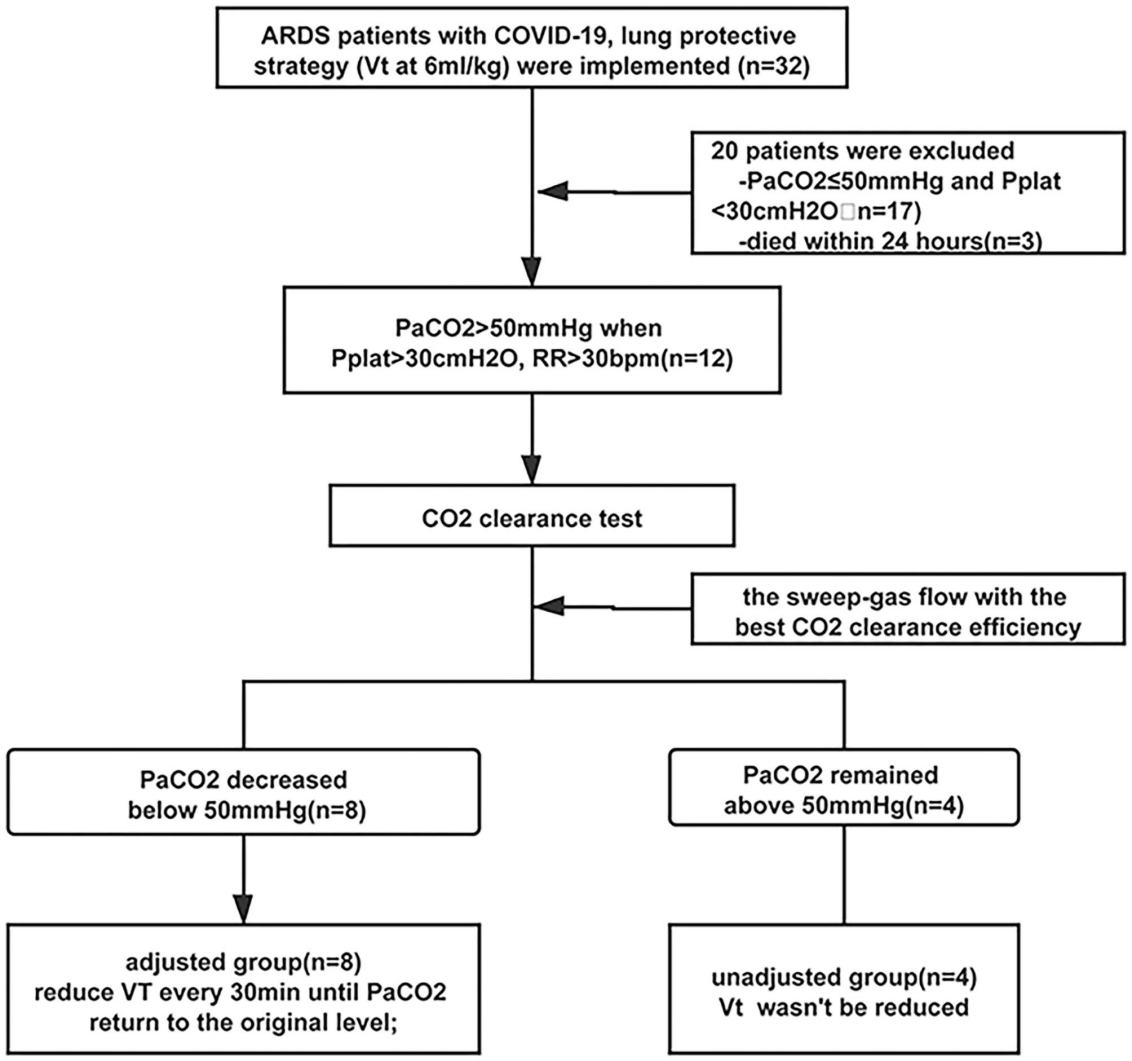
Flowchart of the study.

Half an hour later after, according to the arterial PaCO_2_, the patients were divided into two groups. If the PaCO_2_ decreased to lower than 50 mmHg, VT was gradually reduced from 6 to 5, 4.5 every 30 min until the PaCO_2_ returned to the original level and the pH > 7.2. If the PaCO_2_ still remained above 50 mmHg, the VT would not be changed and the ECCO_2_R device was only used to reduce the hypercapnia. Refractory hypoxemia and/or hypercapnia could be managed at the attending physician's discretion, with recruitment maneuver, prone positioning, and so on. The flowchart is shown in Figure 2.

### Data Collection

Ventilator settings (VT, PEEP, RR, Pplat, minutes ventilation, and FiO_2_), hemodynamic parameters (MAP, HR, and vasopressor dose), arterial blood-gas values (pH, PaO_2_, PaCO_2_, HCO^3−^, and lactate), heparin dose, and aPTTr were collected at baseline. After the run-in time, 30 min, 6 h, and 24 h after the connection, these values were also collected. Other variables such as complete blood count, liver function, and renal function were obtained daily. Respiratory-system compliance, driving pressure, and the mechanical power were calculated according to the standard formulas.

### Statistical Analyses

Results are expressed as median (IQR) when abnormal distribution, and as mean ± SD when normal distribution, and both *p* < 0.05 defined statistical significance. Statistical analysis was performed using non-parameter analysis in chi-square test for comparison between different time intervals when distributed abnormally and using one-way analysis of variance (ANOVA) for repeated measures, followed by a *post-hoc* test, when distributed normally. Analyses were computed with IBM SPSS, version 23.0.1 (SPSS Inc., Chicago, IL, USA) software.

## Results

Thirty-two patients with ARDS were involved, and lung protective strategy worked in 17 patients with PaCO_2_ <50 mmHg, and three patients died within 24 h after admission. After the adjustment of ventilator parameters, PaCO_2_ was still above 50 mmHg (64.5 [56–88.75] mmHg) in 12 patients and the ECCO_2_R devices were applied. At baseline, all these patients received protective ventilation with VT set at 5.94 ± 0.18 ml/kg PBW and PEEP at 6 [5.25, 8.0] cmH_2_O, the respiratory rate was 32.58 ± 3.55 bpm, and the platform pressure and the driving pressure were 34.08 ± 6.91 mmHg and 27.17 ± 5.98 mmHg (Table 1).

**Table 1:** Baseline characteristics.

**Characteristic**	**Total patients (*n* = 12)**	**Adjusted group (*n* = 8)**	**Unadjusted group (*n* = 4)**
Sex (male/female)	6/6	4/4	2/2
Age (years) (IQR)	67.75 [62.25–71.00]	68.5 [63.75–71.5]	64 [58.25–76.5]
SOFA score, (IQR)	8 (7.0–10.0)	6.9 [6.25–11.0]	8.5 (7.25–9.75)
**Pre-ECCO**_**2**_**R adjuvant therapy**			
Neuromuscular blockade	12	8	4
Prone positioning	7	4	3
Recruitment maneuvers	9	6	3
ECMO	0	0	0
**COVID-19 diagnosis**			
Nucleic acid test (+)	12	8	4
Chest CT results(+)	12	8	4
IgM(+)	7	4	3
IgG(+)	6	3	3
Time from symptom onset to intubation (IQR)	27.1 [21.25–34.75]	21 [6.5–36.25]	31.5 [26.75–38.0]
Time from symptom onset to ECCO_2_R initiation (IQR)	43.5 [32.5–47]	39.0 [31.75–47.75]	46.0 [40.5–48]
**Ventilation variable**			
VT (ml/kg PBW)	5.94 ± 0.18	5.9 ± 0.16	5.93 ± 0.15
RR (bpm)	32.58 ± 3.55	31.25 ± 2.96	35.25 ± 3.4
PEEP(cmH_2_O) (IQR)	6 [5.25–8.0]	6 [5.25–7.5]	7 [4.5–9.5]
Pplat (cmH_2_O)	34.08 ± 6.91	29.88 ± 3.04	42.5 ± 3.42
Driving pressure (cmH2O)	27.17 ± 5.98	23.5 ± 2.72	34.5 ± 2.52
Compliance (ml/cmH2O)	13.29 ± 4.88	16.02 ± 3.42	7.83 ± 0.73
**ABG**			
pH (IQR)	7.33 (7.22–7.41)	7.34 (7.22–7.38)	7.30 (7.21–7.37)
PaO_2_ (mmHg) (IQR)	81 (79.25–91.5)	80.5 (79.0–87.75)	87.0 (80.5–111.5)
PaCO_2_ (mmHg) (IQR)	64.5 [56–88.75]	61 [53.5–64.75]	94 [86.25–100.3]
**Outcome**			
Mechanical ventilation durations (days)	12.5 (7.25–33.5)	21.5 (12.25–36.75)	8.2 (5.3–18.0)
ICU length of stay	21 (15.75–36.25)	20.6 (19.5–38.0)	13.5 (7.5–11)
28-day mortality	8/12	4/8	4/4

**Figure 3:**
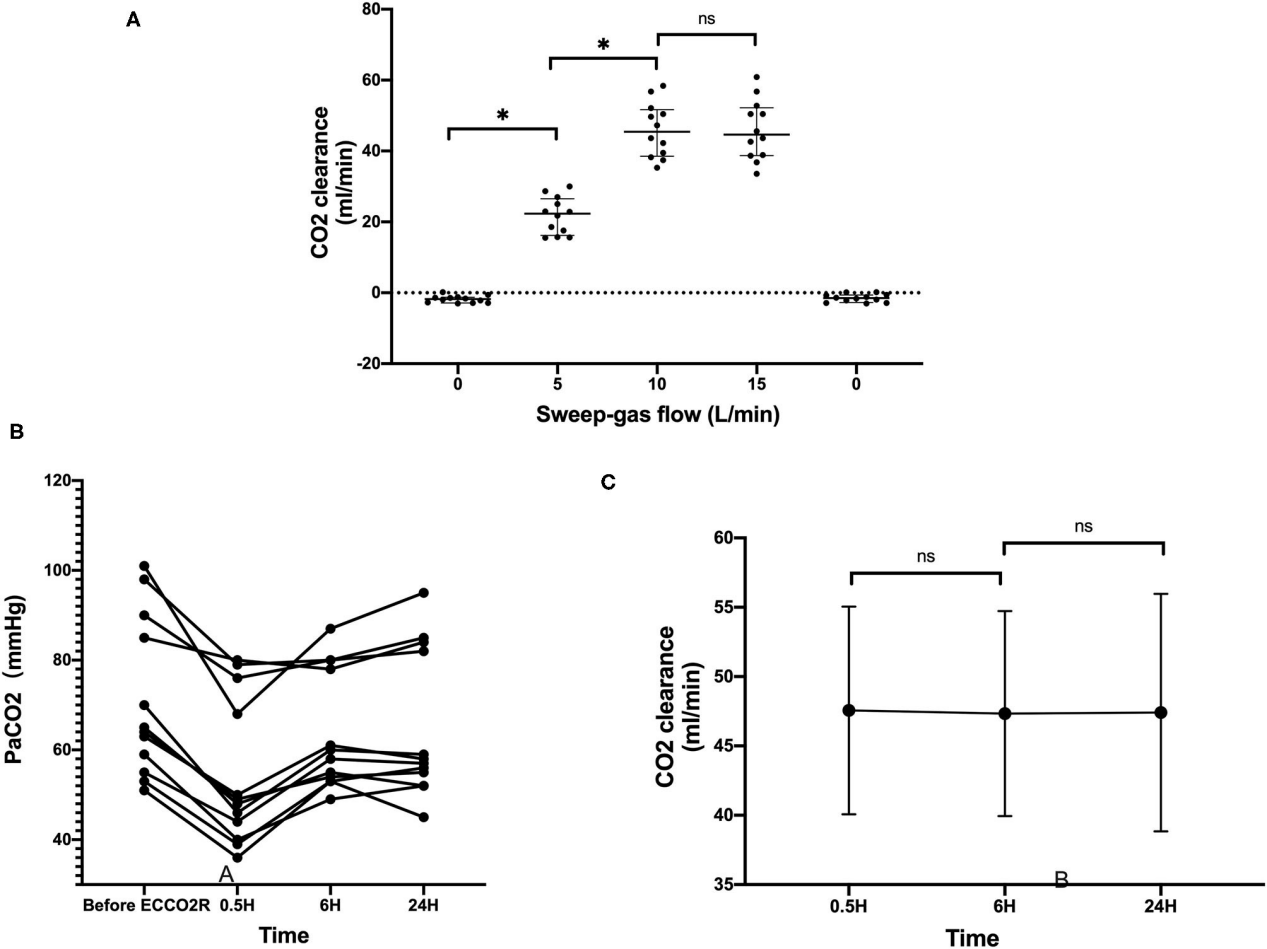
The CO_2_ clearance with the ECCO_2_R. **(A)** The CO_2_ clearance rate at different levels of sweep-gas flow. **(B)** The PaCO_2_ levels of all the 12 patients before and after the ECCO_2_R. **(C)** The CO_2_ clearance rate with time ^*^p < 0.05; ns, no statistical significance.

The mean blood flow was 342.5 ± 49.20 ml/min, and in the flow test, when the sweep-gas flow was set at 10 L/min, the CO_2_ clearance reached the best efficiency, 45.91 ± 7.70 ml/min. In all these patients, the flow was set at 10 L/min during the process. After the application of the ECCO_2_R device, the PaCO_2_ in all the patients decreased, and during the 24 h, the CO_2_ clearance nearly did not change little with time (Figure 3). There was no significant correlation between the CO_2_ clearance and the start PaCO_2_, the DP, and lung compliance.

**Figure 4:**
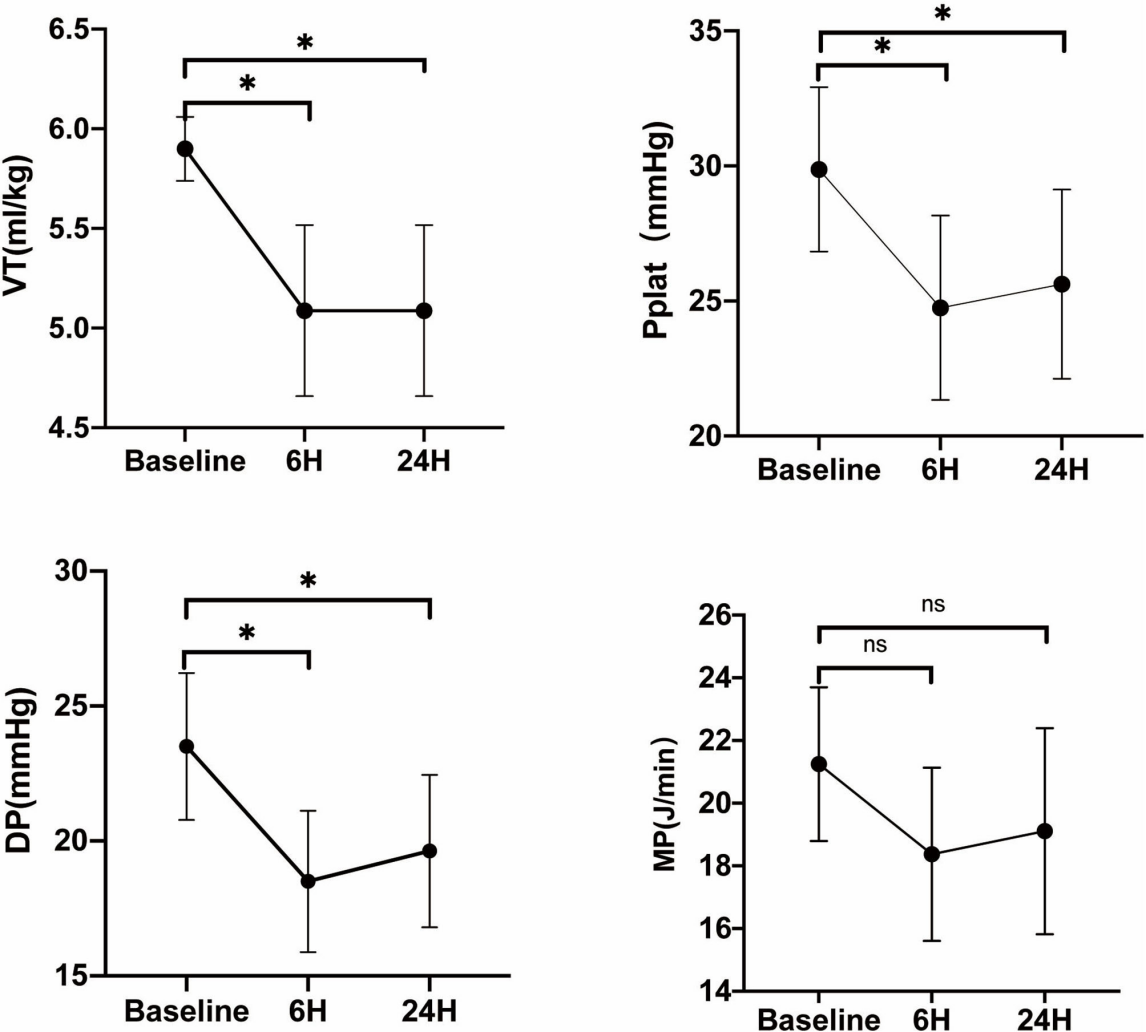
Evaluation of VT, Pplat, DP, and mechanical power when tidal volume was reduced on ECCO_2_R in the adjusted group. Vt, tidal volume; Pplat, end-inspiratory plateau pressure; DP, driving pressure; MP, mechanical power. ^*^p < 0.05 vs. Baseline.

In eight of these patients, the PaCO_2_ could decrease below 50 mmHg, and the VT was reduced every 30 min until the PaCO_2_ returned; in the other four patients, the PaCO_2_ was still above 50 mmHg, and VT was not reduced. According to whether the VT was adjusted, we divided the 12 patients into two groups, adjusted group (*n* = 8) and unadjusted group (*n* = 4). In the adjusted group, 6 h after the flow test, VT was decreased from 5.9 ± 0.16 to 5.08 ± 0.43 ml/kg PBW (*p* < 0.01), and DP and Pplat were also decreased significantly from 23.5 ± 2.72 mmHg and 29.88 ± 3.04 mmHg to 18.5 ± 2.62 mmHg (*p* < 0.01) and 24.75 ± 3.41 mmHg (*p* < 0.01). Furthermore, the mechanical power decreased from 21.25 ± 2.45 to 18.37 ± 2.76 mmHg, with no statistically significant difference (*p* = 0.16). Twenty-four hours later, the DP and Pplat slightly increased, but were still significantly reduced compared with the baseline (Figure 4).

In the unadjusted group, 6 h after the test, PaCO_2_ decreased from 94 [86.25, 100.3] mmHg to 80 [67.50,85.25] mmHg, but with no statistical significant difference (*p* = 0.0571). Twenty-four hours later, the PaCO_2_ increased slightly again.

## Discussion

The result of this single-center, prospective study showed that, in a group of COVID-19 ARDS patients with refractory hypercapnia and extremely low compliance, a low-flow ECCO_2_R system based on the RRT platform can easily and safely reduce the PaCO_2_ level and significantly decrease the Pplat and driving pressure in moderate hypercapnia patients.

Hypercapnia was common with lung protective volume ventilation in COVID-19-related ARDS patients and could be corrected with an intermediate tidal volume (7–8 ml/kg PBW) in some patients (4, 5). The conditions were more severe in our ICU, as 37.5% (12/32) of the ARDS patients had refractory hypercapnia despite ventilated with higher DP and higher respiratory rate than usual, and the lung compliance of our patients were relatively lower than reported in other centers. As in these patients, the hypercapnia occurred in the late stage of this disease in critical patients, which was 43.5 [32.5–47] days after the symptom onset, a reminder that the disease was still in progression at that time. Second, the bilateral diffuse ground-glass opacities and reticulation, compensatory emphysema, architectural distortion, and traction bronchiectasis were typical radiographic features on the CT in severe patients (14, 15), which indicated increased pulmonary dead space in these patients. Last, pathological findings such as exudation and mucous plug with fibrinous exudate in the alveoli could cause ventilatory disfunction.

Although, in the early 1990s, the concept of permissive hypercapnia was proposed for patients with acute lung injury, more studies have reported that hypercapnia has a lot of harmful effects, which include inhibition of cell membrane repair, impairment of alveolar fluid clearance, suppression of innate immunity and host defense (16–18), and significant hemodynamic consequences such as pulmonary hypertension and right ventricular dysfunction (19). Recent data suggest an association between values of PaCO_2_ > 50 mmHg and increased mortality (8); therefore, CO_2_ clearance is a necessary treatment in these patients. The present study showed that CO_2_ clearance could be reached at 45.91 ± 7.70 ml/min, with the low-flow ECCO_2_R device with RRT platform. None of the severe adverse events occurred, although various AEs (e.g., cannulation-related accidents, hemorrhage, pump malfunction, and membrane clotting) were reported (12, 20, 21).

Apart from hypercapnia, the elevated driving pressure and the mechanical power were problems we were more worried about. Because of the low lung compliance of these patients, the driving pressure and the Pplat of these patients were still very high despite protective ventilation with 6 ml/kg. Actually, recent data have demonstrated that there is no safe upper limit for Pplat or DP, and the mortality rate with DP ≤ 14 cmH_2_O is still as far as 20% (9, 22). As the ventilation variable with the best stratified risk, patient outcomes may be improved with the decreasing of DP owing to changes in ventilator settings such as VT (9), and the mechanical power also showed a strong correlation with mortality risk (10). Therefore, in our study, when the PaCO_2_ was reduced with the low-flow ECCO_2_R device, we preferred to decrease the DP and mechanical power first in the moderate hypercapnia group by reducing the VT gradually. In these patients, the DP was significantly reduced, and mechanical power was also reduced, although without statistical significance.

Our results demonstrated that, in these special group of COVID-19 ARDS patients, this low-flow ECCO_2_R system could be easily, safely, and efficiently applied, because the RRT platform is widely available, and it did not require specific venous access. As none of the medications had been proven to be effective in the critical patients with COVID-19, and the ECMO were not adequate in many ICUs (11), this low-flow ECCO_2_R system could provide a new way of correcting the respiratory acidosis and decreasing the DP, apart from the traditional methods such as prone positioning, recruitment maneuver, nitric oxide, and so on. It may help in the effort to reduce mortality in this global campaign against COVID-19.

Several limitations of our work should be addressed. First, only 12 critically ill patients were included. Although this system has been proven to be effective and safe in mild-to-moderate ARDS patients (12), because of the shortage of resources, only patients with the most needs were included, which were the refractory hypercapnia patients. Due to the exploratory nature of the study, which was not driven by formal hypotheses, the sample size calculation was waived. Instead, we hope that the findings present here will encourage a larger cohort study in these special patients. Second, the CO_2_ removal rate of this system was lower than those reported in other studies (23), and the lower blood flows and catheters with faster flow rate could be considered to improve the CO_2_ removal rate. Last, this study was mainly conducted to prove the feasibility of such an ECCO_2_R system applied to refractory hypercapnia patients with COVID-19-associated ARDS, and the system was limited to a period of 24 h, as there was a theoretical risk of rupture of the circuit, and the influence of the outcome of these patients was limited.

## Conclusion

We reported a group of COVID-19 ARDS patients with refractory hypercapnia and extremely low compliance and have demonstrated that a low-flow ECCO_2_R system based on the RRT platform enabled CO_2_ removal and could also decrease the DP and Pplat significantly. This less-invasive ECCO_2_R technique was easily and safely implemented and provided a new way for intensivists in the global campaign against COVID-19.

## Data Availability Statement

The raw data supporting the conclusions of this article will be made available by the authors, without undue reservation.

## Ethics Statement

Ethical review and approval was not required for the study on human participants in accordance with the local legislation and institutional requirements. The patients/participants provided their written informed consent to participate in this study.

## Author Contributions

XZ and YL: study conceptualization and writing—review and editing. XD, HC, HuZ, HoZ, and HH: study design. XD, HC, HuZ, HoZ, HH, WC, CW, WJ, YQ, ZL, JW, XY, TL, and SZ: methodology. XD, HC, HuZ, and HoZ: formal analysis and investigation. XD, HC, and HuZ: writing—original draft preparation. All authors commented on a previous version of the manuscript and all authors read and approved the final manuscript.

## Conflict of Interest

The authors declare that the research was conducted in the absence of any commercial or financial relationships that could be construed as a potential conflict of interest.
